# 3D-printed swab with cover for precision diagnosis

**DOI:** 10.1007/s10856-021-06635-2

**Published:** 2022-01-04

**Authors:** Fan Huang, Kewei Song, Yue Jiang, Kayo Hirose, Shinjiro Umezu

**Affiliations:** 1 Department of Modern Mechanical Engineering, Waseda University, Tokyo, Japan; 2 Department of Anesthesiology and Pain Relief Center, The University of Tokyo Hospital, Tokyo, Japan

## Abstract

The collection capacity of common nasopharyngeal swabs and irregularities of medical personnel limit the accuracy of PCR testing. This study describes a newly designed 3D-printed swab that is combined with a 3D-printed cover to prevent the extraction of undesired nasal secretions. This swab improved the accuracy of PCR test results. The results of a series of experiments showed that, because of the mucus extraction effect, 3D-printed swabs can replace ordinary cotton swabs. The crisis of the worldwide medical supply shortage can be ameliorated to a certain extent by applying 3D printing technology.

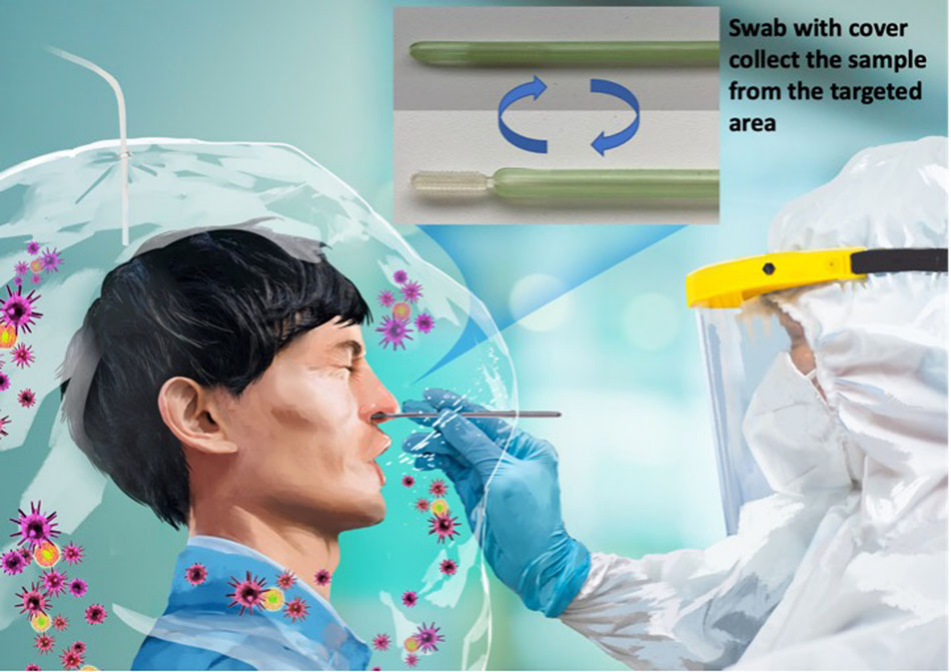

## Introduction

The COVID-19 pandemic has resulted in a global shortage of nasopharyngeal swabs needed for sample collection. This shortage may have an adverse effect by delaying testing for COVID-19 and other respiratory diseases [1, 2]. SARS-CoV-2 can be transmitted by infected individuals who present as only mildly symptomatic or asymptomatic carriers [3]. At the same time, another issue has emerged that has caught the attention of all concerned: the false-negative rate of RT-PCR for COVID-19 is as high as 41% [4]. In the diagnostic process, more than 60% of result errors occur in the pre-analytical phase [5]. When obtaining samples for PCR tests, healthcare personnel may be afraid of being infected; this can lead to mental stress and performance irregularities. This factor, coupled with a lack of a standard swabbing practice, may contribute to the high false-negative rate.

Of the two most common testing instruments currently available, nasopharyngeal swabs have demonstrated a higher viral load than oropharyngeal swabs [6]. Normal nasopharyngeal swabs for diagnostic sampling limit the PCR testing capability [7, 8]. To mitigate a possible future crisis and high false-negative result, using 3D printing technology to produce designed nasopharyngeal swabs is important. In addition, 3D printing enables rapid adjustment of production frequencies depending on the period and region. This approach reduces warehouse floor space and ensures stable production in times of emergency.

Various studies have shown that 3D-printed swabs can be combined with various transport media (including widely available normal saline) without markedly affecting SARS-CoV-2 detection by RT-PCR [9–11]. Callahan et al.’s research has also shown that 3D-printed swabs can efficiently and effectively contribute to resolving a medical manufacturing crisis during a major pandemic [8].

Swabs printed on a 3D printer differ from commercially produced swabs in that 3D-printed swabs have plastic heads that are larger than the heads of cotton or polyester fiber-tipped swabs. The discomfort caused by swabs is intense. According to research [10], epistaxis occurred immediately or shortly following the removal of the swab in 5.0% of employees tested with a 3D-printed swab and in 8.3% of employees tested with a commercial swab. Other minor adverse effects included nasal discomfort, headache, earache, and rhinorrhea, which typically lasted for hours to a day. 3D-printed swabs with a cover are needed for improved patient safety.

Another focus of the present study is the use of a cover to prevent the swab from absorbing virus-less secretions before the swab reaches the nasopharynx. Theoretically, nucleic acid testing is the earliest method to confirm the diagnosis of coronavirus infection (SARS-CoV-2), and methods to ensure the timeliness and accuracy of nucleic acid test results are important to the current epidemic prevention and control [12].

Medical personnel are under tremendous pressure regarding concerns of infection, and false-negative test results from individual samples can occur because of incomplete sampling [13].

The objective of the present study was to develop an accurate 3D-printed nasopharyngeal swab to collect the sample from the targeted area.

## Materials and methods

In the present study, printing was carried out using stereolithography appearance (SLA) technology and resin + photoinitiator as the source material. A plant-based resin was used to make the cover and swab of the COVID testing set. Clear plant-based UV resin was purchased from Anycubic, which has complied with the EN 71-3:2013 safety standard. Plant-based UV resin is advertised as a biodegradable material made primarily from soybean oil. It is a copolymer widely used in 3D printing; it is not caustic but still prints very well, resulting in prints with no discernible difference in quality from regular UV prints. It contains no volatile organic compounds or bisphenol A, which are both common in other industrial resins.

Sample retention of the 3D-printed swab was assessed by the volume, porosity density, and void fraction of the head as well as by the pore geometry. In subsequent design iterations, the tip geometry was optimized for maximum mucus collection. In addition, cover geometry was developed for flexibility, safety, and ease of use. Finally, the tip geometry was ensured to obtain sufficient material.

**Fig. 1:**
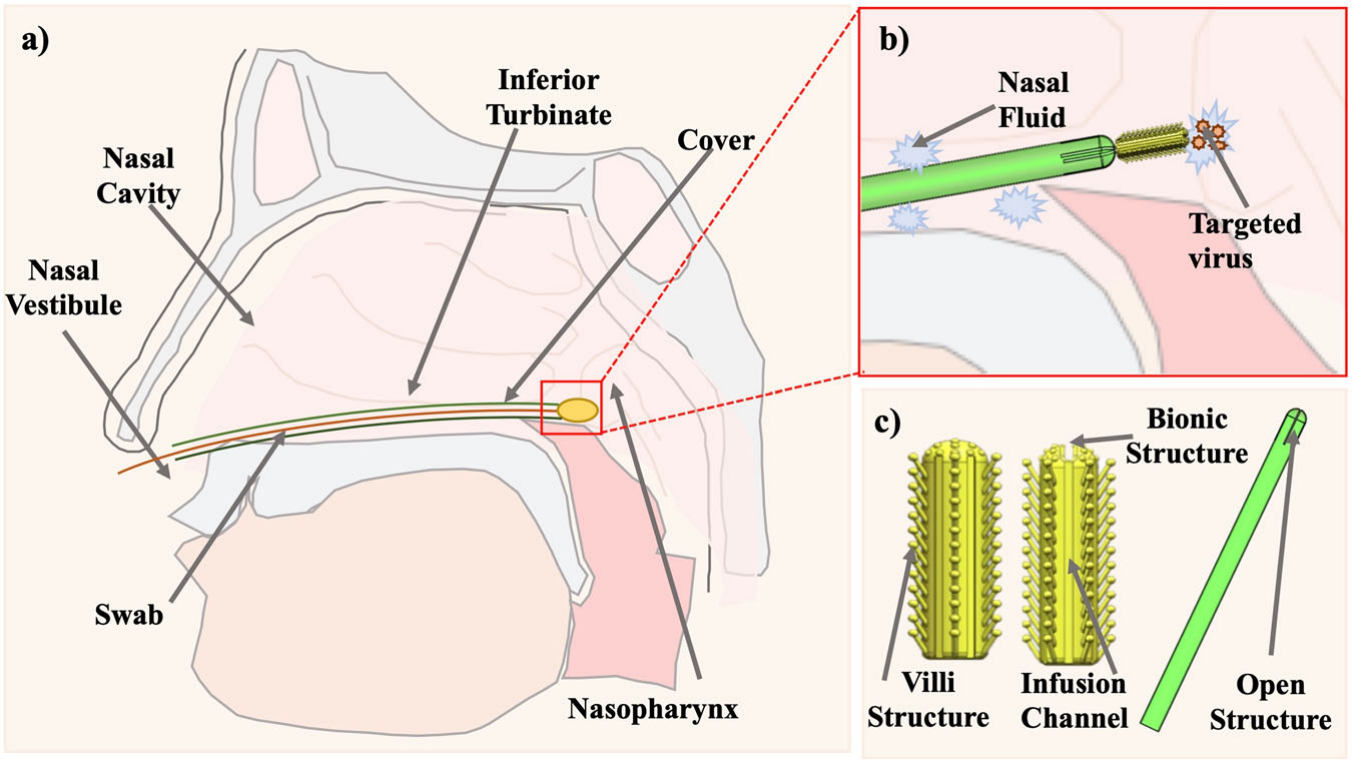
Schematic of nasopharyngeal swab testing for coronavirus. **a** A schema of the pharynx. **b** Partial schematic during collection. **c** 3D-printed swab and cover

Figure 1 shows a schematic of the swab developed in the present study. To obtain the sample needed for the PCR test, the combined cover and swab are simultaneously inserted horizontally into the nasal cavity. When the posterior end of the nasal cavity is reached, the cover is held stationary and the swab is pushed forward to reach the nasopharynx and rotated three to four times to extract the target mucus.

**Fig. 2:**
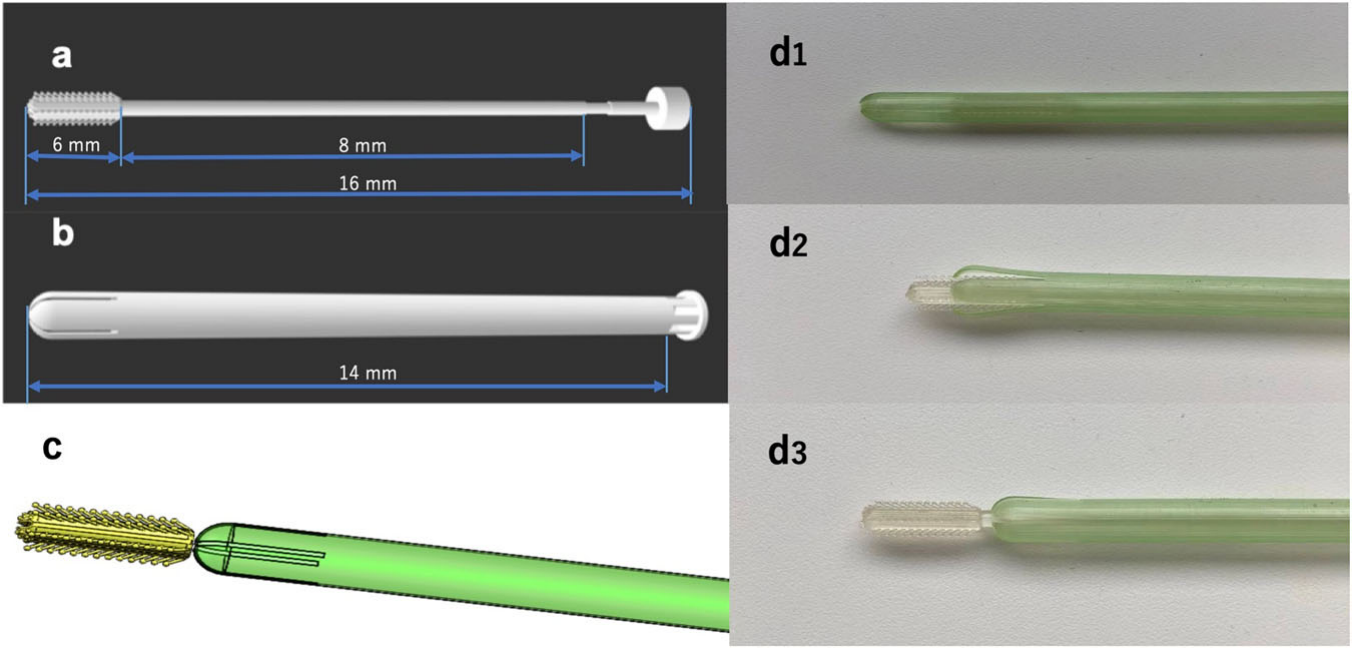
Design of 3D-printed swab kits. **a** Side profile of the swab and cover. **b** Combination of the cover and swab. **c** CAD image of the combination (cover and swab): **d1** before extension; **d2** during extension; **d3** after complete extension

Figure 2 presents an example of a realized nasopharyngeal swab with cover. The basic geometry is composed of a porous head, a holding body, and a breaking point. The inflow trench around the tip enables mucus to flow downward and cover the whole tip, maximizing the mucus contact area for better specimen collection. The diameter and length of the head are 6 and 0.5 mm, respectively. The holding body has a diameter of 10 mm, and the thin breaking point has a diameter of 0.2 mm. The front part of the swab can be broken after the collection of the sample and capped for transport to the testing laboratory. The distance between the end of the swab and the breaking point is 8 mm.

The diameter and length of the cover are 0.6 and 14 mm, respectively. The cover is 0.1 mm thick and is designed this way for two reasons. With the same inner diameter, when the cover is thicker, the diameter of the swab will be larger; thus, the patient is more likely to feel discomfort. In the opposite case, when the cover is too thin, it becomes fragile because it is difficult to fabricate by 3D printing and its mechanical strength is reduced. For these reasons, the thickness of the cover was set to 0.1 mm in the present study. The bottom of the cover is designed for the cover to be easily removed from the printer platform. Figure 2c shows the combination of the cover and swab. The whole extension process is shown in Fig. 2d1–3. During the extension process, the cover is in the open configuration and the swab can be pushed out of the cover and touch the nasopharynx area. When the swab has been completely pushed out, the cover will return to the semi-closed configuration. After sampling, the swab can be withdrawn into the cover again.

Artificial mucus model: polyvinyl alcohol was mixed with 300 mL Milli Q water to achieve a final viscosity of 200 mPa·s. In healthy subjects, large deviations are observed in nasal mucus. Nasal mucus from healthy subjects exhibits a viscosity of 1.3 Pa·s at a shear frequency of 100 rad/s [14]. According to the measurement range and shear frequency of the laboratory viscometer, an artificial mucus model with a viscosity of 200 mPa·s was prepared.

## Results

Structural performance analysis of this design was processed using finite element analysis. The solver of choice to mimic a bulking and compression test was Abaqus/CAE 2019. The element type used for Abaqus was C3D8R, which is a general-purpose linear brick element, with reduced integration. Swabs must exhibit good mechanical behavior; they are expected to maintain an undeformed shape in compression tests and a good toughness to resist plastic deformation in buckling tests.

To reduce the calculation load and focus on the desired properties, the swab was divided into two parts for analysis: the head part and the rod part. The head part was modeled in the compression test, and the rod part was modeled in the buckling test (Table 1).

**Table 1:** Material properties of the Anycubic translucent UV resin

Material	Value
Tensile modulus	2500 M Pa
Tensile strength	50 M Pa
Poisson’s ratio	0.4
Density	1.15 g/cm^3^

**Fig. 3:**
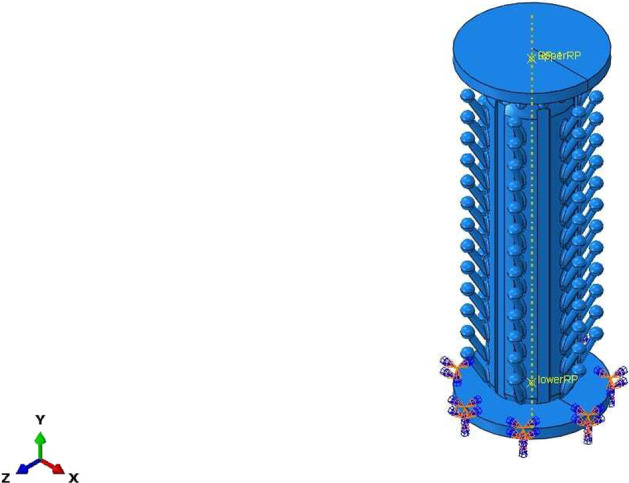
Boundary conditions and loads applied to the swab

The compression test simulation consisted of three parts: two platens with a diameter and thickness of 6 and 0.5 mm, respectively, as rigid bodies and one swab head. One platen was pressed onto the top of the head part, and another platen was set at the bottom of the head part. A 1 N vertical load was applied at the center point of the top platen along the −*y* direction (in-house tests were carried out on commercially available traditional swabs, which showed plastic deformation between approximately 0.8 and 1.2 N) [15]. In addition, a velocity of 22.5 mm/s was applied to the whole top platen along with −*y* direction because the swab entry process takes 4 s to move through 9 cm of the nasal cavity to reach the nasopharynx. The platen at the bottom was assigned as fixed in all directions, as shown in Fig. 3.

**Fig. 4:**
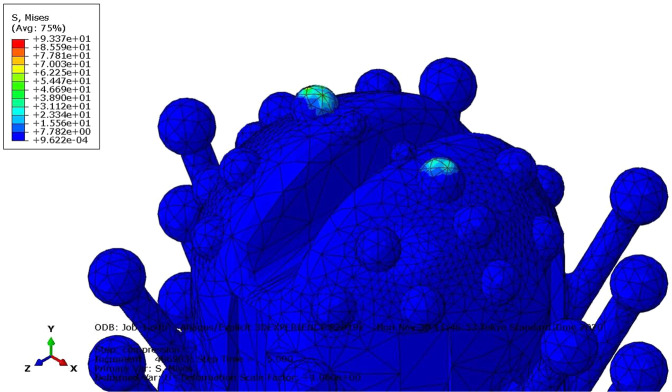
Deformed swab and von Mises stress profile

Figure 3 shows the boundary conditions and loads applied to this model. The process of applying load and velocity to the tip of the swab vertically was modeled in Abaqus/CAE 2019 to mimic the resistance of the nasopharynx during the sample collection. This model was then submitted to Abaqus/Explicit Solver to solve. The contour plot was obtained as shown in Fig. 4. The maximum von Mises stress is ~93 MPa at the tip of the head part. Compared with its original shape shown in Fig. 3, the model showed no obvious deformation during the entry process. The elongation at break is approximately 11–17%; hence, this kind of newly designed swab is not likely to be broken in this specific situation. In this way, a safe testing procedure that causes little pain to the patient could be achieved.

**Fig. 5:**
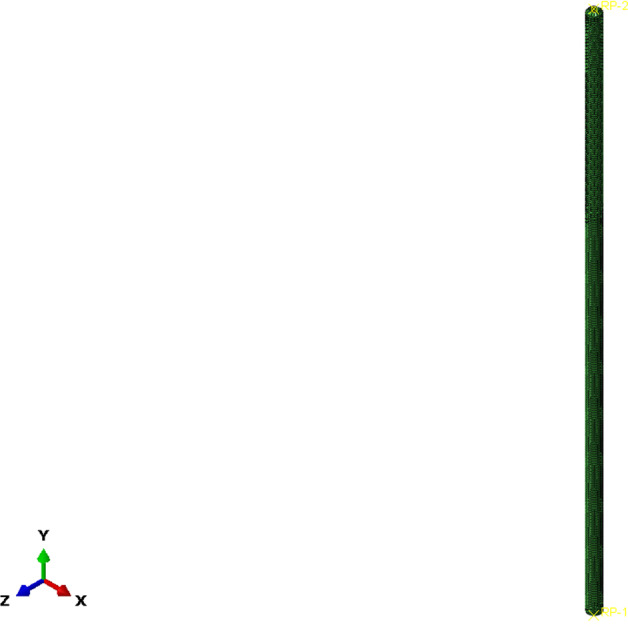
Buckling test simulation of the rod

The rod part of the swab was selected for the buckling test simulation. The top part of the rod is fixed in all directions with 0 displacement. A 50-mm displacement was applied (because the total length of the rod was 100 mm) at the bottom of the rod vertically along with the *y* direction, shown in Fig. 5.

**Fig. 6:**
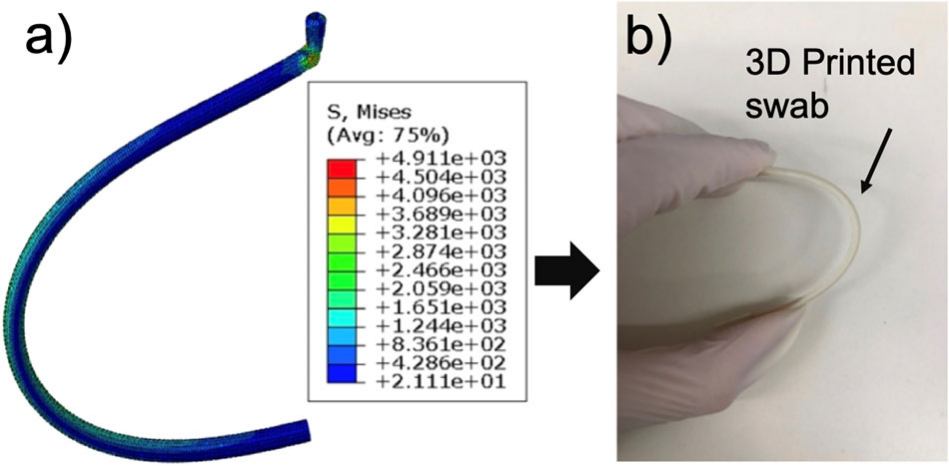
Schematics. **a** Contour plot of the rod. **b** An actual bending test

Figure 6 shows the contour plot obtained from the swab rod buckling test simulation. It shows that the rod deformed without damage to the bending area and that the von Mises stress in the bending area was approximately 1600–2000 MPa. The simulation result shows that the newly designed swab made of UV resin exhibits sufficient strength to resist plastic deformation, consistent with the results of the actual experiments.

The photo on the right side of Fig. 6 shows the bending test, which was performed several times. The aim was to check the swab’s robustness to fracture, the neck’s flexibility and robustness to fracture, and the robustness to repeated insertion into and removal from a tortuous canal (diameter, 3 cm). The results show that the 3D-printed swab can tolerate rough repeated insertions into a 1-cm diameter clear plastic tube that curves back on itself. The neck can be slowly and repeatedly bent 180° without breaking.

**Fig. 7:**
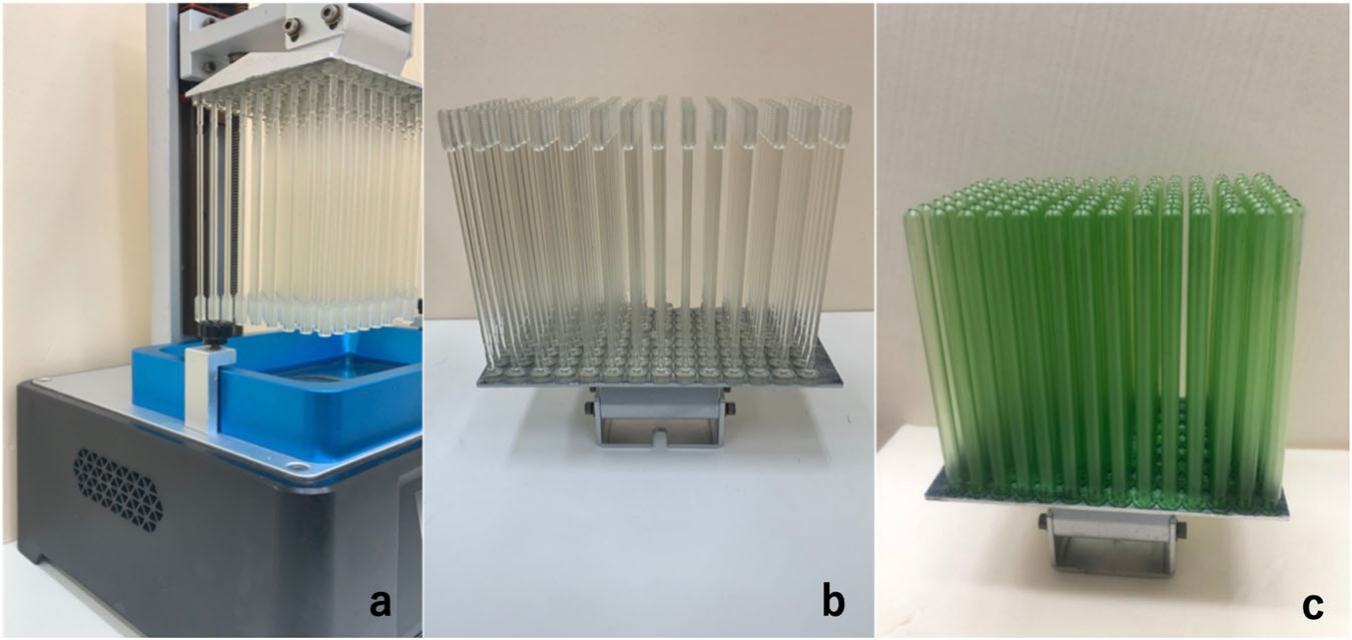
Practical production photos. **a** Printing process. **b** Printing result of the swabs. **c** Printing result of the covers

As shown in Fig. 7, a single SLA printer can mass produce 100 swabs simultaneously. A resin volume of 66.31 mL is needed. The entire printing process requires 5 h and 12 min to finish. All swabs were cleaned with 70% alcohol for sterilization and light-cured before all tests.

**Fig. 8:**
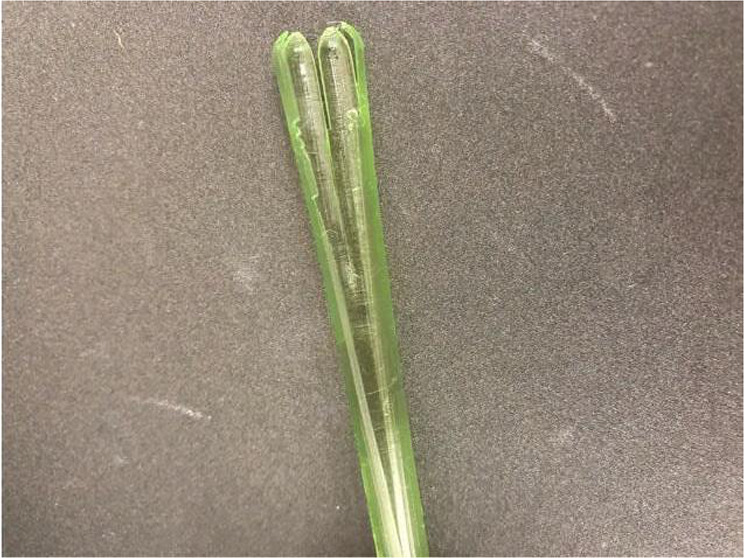
Cover crack area, with a soft edge

A simulated damage experiment demonstrated the safety of the 3D-printed cover, as shown in Fig. 8. On the basis of computed tomography measurements of an asymptomatic adult-sized nasal air space, the nasal cavity with an inner diameter of 6.35 mm is larger than the cover with an inner diameter of 6.00 mm. Therefore, intended use will not result in the breaking of the cover. If use is not correct and leads to cracks, the test results show that the edges of the cracks will be soft and that no pieces will break off in the nasal cavity. The patient’s nasal cavity will not be scratched or punctured.

**Fig. 9:**
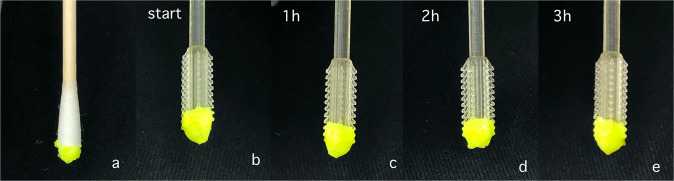
PCR compatibility test. **a** Result for a normal cotton swab dipped into target mucus. **b** Result of a 3D-printed swab dipped into target mucus. **c** 3D-printed swab after 1 h. **d** 3D-printed swab after 2 h. **e** 3D-printed swab after 3 h

To assess the swab’s ability to retain sufficient material, we tested it by placing it head-downward after breaking it off at the breakpoint. Normal cotton swabs were tested for comparison. According to the recommendations issued by the National Health Care Commission of China, samples should be sent for testing within 4 h after collection [16]. Thus, for both 3D-printed swabs and normal swabs, the maximum inverted resting time should be approximately 3 h. The PCR compatibility test results are shown in Fig. 9. After 3 h, no mucus droplets dripped from the designed tip or the normal tip. Therefore, the 3D-printed swabs showed a good ability to retain sufficient material in the inverted state, demonstrating that they could replace ordinary cotton swabs for practical use.

**Fig. 10:**
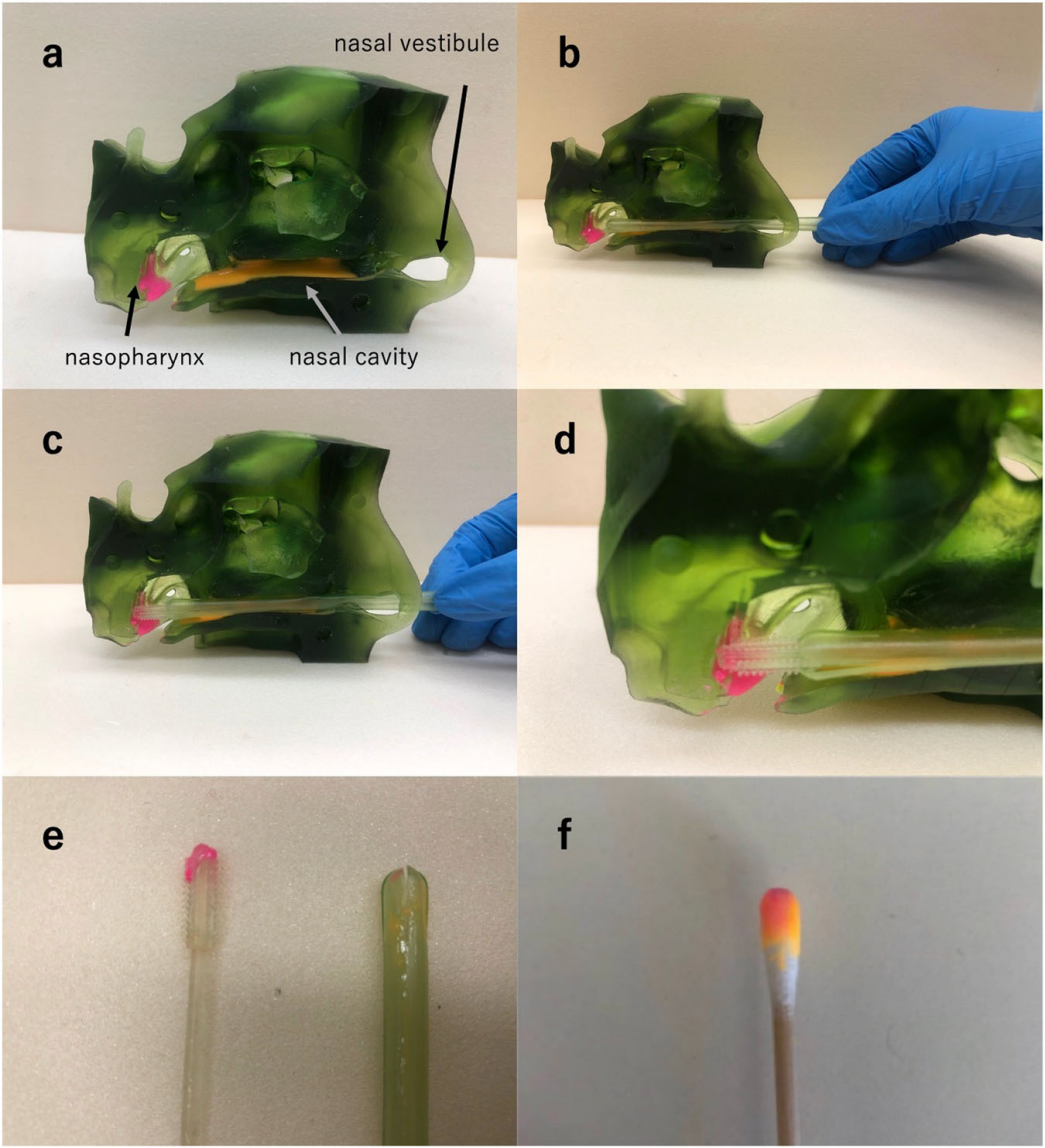
Collection process of mucus-like material. **a** Applied mucus (pink is artificial mucus representing target nasal secretions; orange is artificial mucus representing non-target nasal secretions with fewer virus particles). **b** The 3D-printed swab with cover is horizontally inserted. **c** The swab is inserted further to extract mucus from the nasopharynx. **d** Partial detail of the nasopharynx. **e** The 3D-printed swab tip only picked up pink-colored mucus, and the cover has orange- and pink-colored mucus on the surface. **f** The normal cotton swab tip picked up both orange- and pink-colored mucus

A simulation of nasopharyngeal swab collection (shown in Fig. 10a) was performed as described in Nicolas’s study [17]. The simulation allows us to conduct a realistic nasopharyngeal swab collection. This tool was used to test if the cover can prevent the swab from absorbing virus-less secretions.

Using the simulator, the swab kit (combination of cover and swab) can be inserted through the nostril, parallel to the palate, and gently advanced horizontally to the nasopharynx. After collection, the swab can be withdrawn in the same axis. The nasopharynx is where the pink-colored mucus is located. Around the nostril, orange-colored mucus has been placed to represent virus-less mucus. After the withdrawal of the swab, only the target sample was present on the tip (Fig. 10e). When ordinary cotton swabs were used as a control and the same procedure was performed, the tip contained both types of mucus (Fig. 10f). These experiments show that, compared to the commonly used swab, the 3D-printed swab kit is effective for avoiding non-target mucus and extracting only target mucus (electronic supplementary material, Movie 1). In addition, the cover prevented the holding body from contacting any mucus. After a PCR test, medical personnel can cut the swab tip down and deliver the part to a testing center. Thus, delivery efficiency can be improved because the tip occupies little space.

## Discussion

This study showed that a designed 3D-printed swab with a 3D-printed cover collects targeted mucus separate from the aspect of the nasal cavity in a horizontal direction. In addition to the remarkably higher accuracy of the 3D-printed swab in comparison with a normal cotton swab, the designed swabs are shown to be advantageous with respect to the efficiency of production and reliable quality. How the targeted mucus varies on the swab tip spatially and temporally was also demonstrated. The primary metric for comparing the efficacy of swabs is how long they maintain the mucus. The results presented here confirm the findings of Callahan et al. [8] that 3D-printed swabs are a good alternative to normal cotton swabs.

A limitation of the experimental setup is that it tested only one adult nose manikin. Hence, these results do not allow a conclusion that the 3D-printed swabs are safe for children. However, because of the flexibility of 3D printing technology, the 3D-printed swabs and covers can be resized and produced within a short time.

The production speed of the 3D-printed swab is closely related to the number of available printers. A low production speed will weaken the production advantages of 3D printing. The number of 3D printers can be adjusted in advance by estimating the demand for swabs in each period to ensure a stable production output.

The initial application of 3D printing technology in the medical field has overturned the perception of existing medical treatments. Many developed countries have invested sufficient funds to make 3D printing a global strategy for human health. Regenerative medicine tissue engineering based on 3D printing technology has become one of the most active research areas in the life sciences, with emphasis currently placed on the technological competition after the completion of large-scale sequencing of human genes.

The China Food and Drug Administration (CFDA) has issued new guidance with registration requirements for devices produced through 3D printing or additive manufacturing. In 2016, the US Food and Drug Administration (FDA) issued draft guidance to provide recommendations for manufacturers of devices produced through 3D printing technology.

In the future, with CFDA- and FDA-supportive policies for medical 3D printing and the growing maturity of technology applications, medical 3D printing companies involved in the 3D printing of medical devices may usher in a capital boom.

## Conclusion

In this study, we reported on the safety and acceptability of 3D-printed swabs with covers for collecting mucus samples for SARS-CoV-2 testing. Finite element analysis was used to evaluate the mechanical performance of the designed swabs and covers.

This work builds on recent work in the United States, where 3D-printed swabs are being evaluated. The results showed that the addition of a cover has the advantages of maintaining the safety of PCR testing and enabling the accurate extraction of viruses located in the nasopharynx.

Compared with other hollow-structure designs, the tip design in this study takes more volumetric space, thus reducing the number of swabs that can be printed in a batch when using SLA.

Because of the widespread availability of 3D printing technology, most developed and developing countries can ensure a local adequate swab supply. According to different situations and demands, swabs can be easily designed and produced in a short time. The scalability of this new production method means that thousands of designed swabs can be produced and transported each day. The possibility of producing swabs on demand can effectively reduce waste and inventory. The high false-negative rate of current testing methods has highlighted how 3D printing can lead to a healthier and more environmentally friendly future.

## Supplementary Information

Movie 1
